# Myofibroblastic stromal reaction and lymph node status in invasive breast carcinoma: possible role of the TGF-β1/TGF-βR1 pathway

**DOI:** 10.1186/1471-2407-14-499

**Published:** 2014-07-09

**Authors:** Xavier Catteau, Philippe Simon, Jean-Christophe Noël

**Affiliations:** 1 Department of Pathology, Institute of Pathology and Genetics, 25, Avenue Georges Lemaître, Gosselies 6041, Belgium; 2 Faculty of Medicine, Université Libre de Bruxelles, Brussels, Belgium; 3 Gynecology Unit, Erasme University Hospital-Université Libre de Bruxelles, Brussels, Belgium; 4 Gynecopathology Unit, Pathology Department, Erasme University Hospital-Université Libre de Bruxelles, Brussels, Belgium

**Keywords:** Breast carcinoma, Tumor microenvironment, Fibrocytes, Myofibroblasts, SMA, CD34, TGF-ß, Metastasis, Lymph node

## Abstract

**Background:**

The microenvironment modulates tissue specificity in the normal breast and in breast cancer. The stromal loss of CD34 expression and acquisition of SMA myofibroblastic features may constitute a prerequisite for tumor invasiveness in breast carcinoma. The aim of the present study is to examine the stromal expression of CD34 and SMA in cases of invasive ductal carcinoma and to try to demonstrate the role played by the TGF-ß 1 et TGF-ß R1 pathway in the transformation of normal breast fibrocytes into myofibroblasts.

**Methods:**

We carried out an immunohistochemical study of CD34, SMA, TGF-ß and TGF-ß R1 on a series of 155 patients with invasive ductal carcinoma. We also treated a breast fibrocytes cell line with TGF-ß1.

**Results:**

We found a loss of stromal expression of CD34 with the appearance of a myofibroblastic reaction in almost 100% cases of invasive ductal carcinoma. The strong stromal expression of SMA correlates with the presence of lymph node metastases. We were also able to show a greater expression of TGF-ß in the tumor cells as well as a higher expression of TGF- ß R1 in the tumor stroma compared to normal breast tissue. Finally, we demonstrated the transformation of breast fibrocytes into SMA positive myofibroblasts after being treated with TGF-ß1.

**Conclusions:**

Our study demonstrated that a significant tumor myofibroblastic reaction is correlated with the presence of lymph node metastasis and that this myofibroblastic reaction can be induced by TGF-ß1. Future research on fibrocytes, myofibroblasts, TGF-ß and stromal changes mechanisms is essential in the future and may potentially lead to new treatment approaches.

## Background

Breast cancer is the most common cancer among women in the world [1]. The microenvironment modulates normal breast tissue, as well as the growth, survival, polarity, and invasive behavior of breast cancer cells [2,3]. The stromal loss of CD34 expression and acquisition of smooth muscle actin (SMA) myofibroblastic features may constitute a prerequisite for tumor invasiveness in breast carcinoma [4,5]. The origin of myofibroblasts is not yet clear and multiple hypotheses have been proposed. Myofibroblasts modulate the stroma in physiology and pathology through direct cell-to-cell contact and through secretion of different proteinases, extracellular matrix (ECM) components, growth factors and cytokines. Transforming Growth-Beta (TGF-ß) are multifunctional cytokines which inhibit epithelial cell growth, stimulate mesenchymal cell proliferation, regulate ECM, modulate immune function and wound repair. A desmoplastic reaction is frequent in many solid tumors, such as breast tumors, in which high levels of TGF-ß are found [6-9]. Casey et al. demonstrated that TGF-ß1 treatment in vitro activates normal primary breast fibroblasts and carcinoma-associated fibroblasts (CAFs) into myofibroblasts [10,11], and subcutaneous injections of TGF-ß1 into mice stimulates the formation of reactive stroma [12]. We hypothesize that TGF-ß facilitates breast cancer invasion by stimulating the appearance of myofibroblasts and creates an environment that promotes invasion and facilitates metastasis. The present study aims to investigate this phenomenon in cases of invasive ductal carcinoma (IDC) and to try to understand the underlying mechanism responsible for this myofibroblastic reaction, especially the role played by TGF-ß.

## Methods

### Study population

Breast tissue from cancer patients and normal controls (reduction mammoplasty) was collected from consecutive patients who were identified through the Pathology and Genetics Institute (IPG), resulting in 165 consecutive patients diagnosed between January 2010 and December 2012. This retrospective study was performed on 155 cases of invasive breast carcinoma and 10 cases of reduction mammoplasty from normal breast tissue to compare the expression of the antibodies between the tumor and normal breast tissue. All patients were female. 83 resection specimens and 82 biopsies were obtained. The study protocol was approved by the institutional ethics and research review boards at Erasme Hospital. People sign a written informed consent on admission to the hospital. Consent requires that physicians have the right to use the surplus biological material. The material that has not been used for diagnosis can be used for research (opting out system). Consent has been established by the local ethics committee and is in accordance with Belgian and International law (Helsinki declaration). The final pathological tumor stage was determined using the TNM staging system (AJCC Cancer Staging Manual, 7th edition, 2007) and graded using the Nottingham system [13]. In addition, the patient’s age, tumor size, tumor shape, estrogen receptor (ER), progesterone receptor (PR), HER2/Neu status and KI-67 index were assessed in per cases. Among them, radiologists reviewed the radiological images of tumors and classified them as nodular, spiculate or mixed lesions.

### Cell line cultures

Human mammary fibrocytes P10893 was purchased from Innoprot® and maintained in Innoprot-recommended media and conditions. The media were changed every two days. When cells reached confluence they were passaged to a 25 cm^2^ flask (Corning® Plasticware Cell Culture, Corning, NY, USA) by treating with 0.25% trypsin-25 mM EDTA (Gibco® Invitrogen Corporation) and agitating until cells began to detach from the surface of the flask (passage 1; p1). P2 cells were moved to a 75 cm^2^ flask and then passaged 1:4. All experiments were performed on fibrocytes that had been cultured for 3–10 passages.

Cells were phenotypically characterized by immunostaining. Cells positive for vimentin and negative for cytokeratin staining were considered fibroblasts. Cells were plated in six well chamber slides (Corning®), and grown to confluence. Cells were washed with PBS and fixed with 4% buffered formalin and immunostained according to the manufacturer’s protocols.

### Assessment of fibrocytes activation into myofibroblasts

Cells were plated and grown to confluence in six-chamber slides in basal medium. Media was aspirated from the cultures and cells were washed twice with PBS and then incubated for 24 h in serum-free media with 0 or 2.5 ng/ml TGF-ß1 (Peprotech®) for 48 h with a change in the culture medium after 24 h. After 48 h, cells were fixed, and incubated with SMA and CD34 antibodies. The percentage of myofibroblasts was assessed by counting at least 1,000 total cells and determining the proportion stained positively for SMA in three fields at 200X in duplicate preparations.

### Immunohistochemistry

The specimens were fixed in histology-grade 4% buffered formalin. Series paraffin sections were stained with hematoxylin and eosin and immunohistochemical detection was performed according to the manufacturer’s protocols (Table 1). We used a fully automated immunohistochemical system (Autostainer Link 48 from Dako®).

**Table 1:** Antibodies used in this study

**Antigen**	**Clone**		**Dilution**	**Source**	**Catalog number**
CD 34	QBEnd-10	Monoclonal Mouse	Ready-to-use	Dako	IR63261
Vimentine	V9	Monoclonal Mouse	Ready-to-use	Dako	IR63061
α-SMA	1A4	Monoclonal Mouse	Ready-to-use	Dako	IR00611
CKAE1/AE3	AE1/AE3	Monoclonal Mouse	Ready-to-use	Dako	IR05361
TGF-ß	TGFB17	Monoclonal Mouse	1/20	Vector	VP-T486
TGF-ß R1	8A11	Monoclonal Mouse	1/25	Vector	VP-T487

### Semi-quantitative assessment of immunohistochemistry

We analyzed the stromal distribution of CD34 and SMA in the tumor. Immunohistochemical expression of TGF-ß and transforming growth-Beta receptor-1 (TGF-ßR1) was evaluated in normal breast tissue (glands and stroma) and in tumor tissue (tumor cell and stroma). The immunoreactivity of CD34, SMA, TGF-ß and TFG-ßR1 was assessed semi-quantitatively. The percentage of stromal cells expressing CD34 and SMA was graded as “0”, “+”, “++”, “+++”, “++++” when up to 5%, more than 5% and up to 25%, more than 25% and up to 50%, more than 50% and up to 75% or more than 75% of stromal cells, disclosed immunoreactivity, respectively. Percentages were assessed by two independent observers, assuming that a high-power microscopic field (objective x40, microscopic magnification: x400) harbored 100 stromal cells (range: 75–150). We also evaluated the presence or absence of expression of TGF-ß and TGF-ßR1 in glands and stroma of normal and tumor tissue. Staining intensity for the TGF-ß and TGF-ßR1 antibodies was assessed in a semiquantitative manner by XC and JCN using the H scoring system as described by McCarty et al. [14]. Briefly, scores are generated by adding together 3 ×% strongly staining, 2 ×% moderately staining, and 1 ×% weakly staining, giving a possible range of 0 to 300. An H-score >50 was considered as positive. An assessment of total percentage of cells showing positive staining was also carried out. When disagreements occurred between the two observers they were resolved using a double-headed microscope.

### Statistical analysis

The relationship between the staining patterns of SMA and different clinical and histological features - age, tumor size, tumor shape, grade of invasive carcinoma, lymph node status, luminal classification, and KI-67 index - was compared using a Chi-squared test. A Student’s *t*-test was used to compare H-score and percentage positivity. A p-value <0.05 was considered statistically significant. All analyses were performed using Statistica®.

## Results

### Clinicopathological features of invasive breast carcinoma patients

This study was performed on 155 cases of IDC. All cases were female. Their ages ranged from 25–100 years with a mean age of 61.1 years. Table 2 summarizes the clinical and histological features of the study population. In all cases, the peritumoral stroma appeared fibrous (desmoplastic) upon routine staining with hematoxylin-eosin. This fibrosis appeared to be hyaline in 90% of cases and eosinophilic in 10% of cases. Stromal cells were fusiform, had spindle-shaped nuclei and did not show nuclear-cytoplasmic atypia. 65% (101/155) of tumors had a stellar pattern, 21% (33/155) of tumors a nodular pattern and 14% (21/155) of tumors showed a mixed pattern. When the cells were organized in nodular pattern, the stroma between the cells was less visible but was nevertheless present.

**Table 2:** Clinicopathological data of 155 cases of invasive breast carcinoma

**Clinical data**	**No (%)**
Tumor size	
T1 (0.1- 2 cm)	101 (65)
T2 (>2- 5 cm)	50 (32)
T3 (>5 cm)	4 (3)
Tumor grade*	
Grade 1	32 (21)
Grade 2	84 (54)
Grade 3	39 (25)
Tumor shape	
Spiculated	101 (65)
Nodular	33 (21)
Mixed	21 (14)

*The Nottingham system was used to assess tumour grade.

### Stromal CD34 and SMA in vivo expression

**Figure 1:**
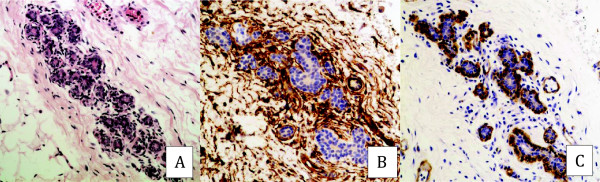
**CD34 and SMA expression in normal breast tissue. A**: normal breast ducts (X100; H&E staining). **B**: diffuse CD34 expression within the periductal stroma of normal ducts (X100). **C**: absence of SMA expression within the periductal stroma of normal ducts (X100).

**Figure 2:**
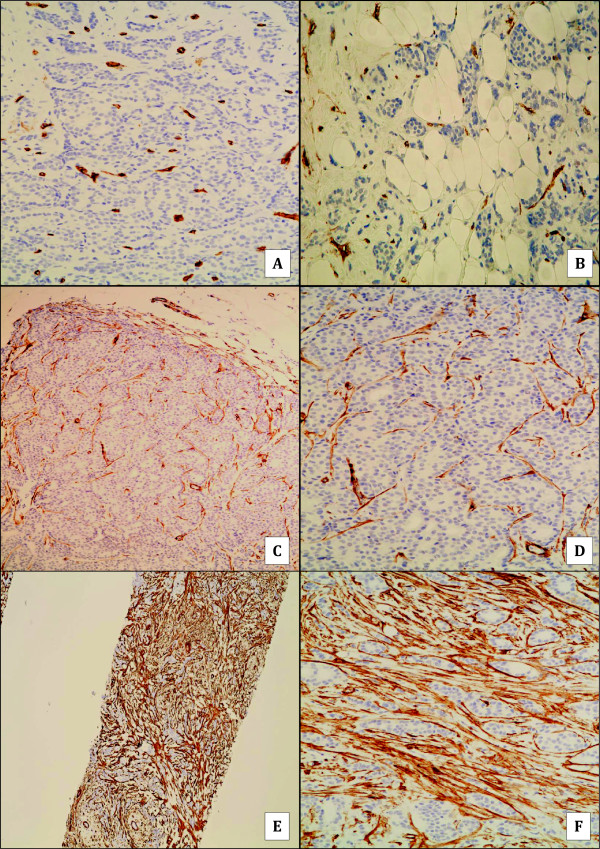
**Stromal CD34 and SMA expression in invasive ductal carcinoma. A** and **B**: absence of stromal expression of CD 34, except within invasive ductal carcinoma vessels (X100). **C** and **D**: example of weak stromal expression of SMA within invasive ductal carcinoma (C:X40; D:X100). **E** and **F**: example of strong stromal expression of SMA within invasive ductal carcinoma (E:X40; F:X200).

In normal mammary tissue, muscular blood vessels, glandular ducts, and acinii were surrounded by a dense concentric network of CD34 fibrocytes. Slight CD34 staining was noted on small-caliber blood vessels within the stroma. No CD34 reactivity was observed in epithelial cells. SMA was detected in the wall of muscular vessels and in the myoepithelia lining the ductal and acinar basement membranes, whereas SMA-reactive myofibroblasts were not detected in the stroma of normal breast tissue (Figure 1). The cancer-associated stromal cells were SMA-positive, vimentin-positive and cytokeratin-negative, confirming their identity as myofibroblasts. Myofibroblasts were found intimately surrounding tumoral cells (Figures 2C to E). In all 155 cases of IDC, the stroma showed a complete loss of CD34 fibrocytes except around the vessels, while the surrounding mammary tumor-free tissue disclosed a normal distribution of this cell population (Figures 2A and B). About 97% (151/155) of IDC revealed SMA myofibroblasts. 78.7% (122/155) showed a significant to very significant myofibroblastic reaction (+++, ++++) while 18.7% (29/155) showed low

to moderate expression (+, ++). There was a significant relationship between SMA and LN status. Strong (+++, ++++) SMA expression was significantly related to the presence of lymph node metastasis (p <0.05). No significant relationship was present between SMA expression and other clinicopathological data (Table 3).

**Table 3:** Relation of stromal expression and clinicopathological features

	Strong expression SMA*	Weak expression SMA**	
Age			
≤ 40	7	1	p = 0.9
>40- ≤ 60	52	14	
> 60	58	16	
Grade			
Grade 1	27	6	p = 0.98
Grade 2	62	18	
Grade 3	33	9	
Tumor shape			
Spiculated	86	20	p = 0.2
Nodular	18	9	
Mixed	18	4	
Tumor size			
≤ 1 cm	28	12	p = 0.1
> 1 - ≤ 2 cm	42	9	
> 2 cm	46	9	
KI-67 index			
≤ 15%	53	14	p = 0.9
> 15%	59	18	
Lymph node status			
Metastasis	52	8	***p < 0.05***
No metastasis	40	16	

Significant P value is <0.05.

*Strong expression means that > 50% of stromal cells express SMA (score +++ and ++++).

**Weak expression means that < 50% of stromal cells express SMA (score + and ++).

### TGF-ß and TGF-ßR1 in vivo expression

**Figure 3:**
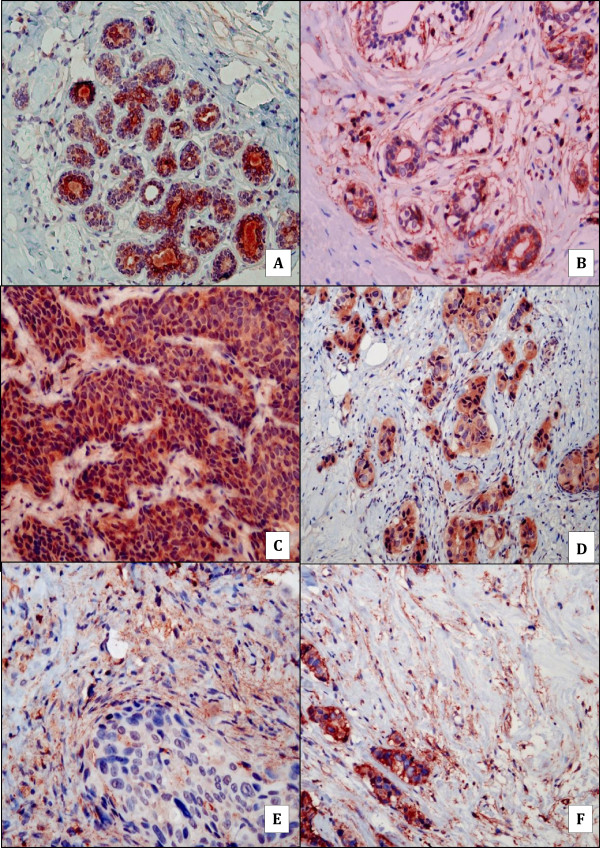
**TGF-ß and TGF-ßR1 expression in normal breast tissue and invasive ductal carcinoma. A**: presence of ductal expression of TGF-ß in normal breast tissue (X100). **B**: presence of ductal expression of TGF-ßR1 in normal breast tissue (X200). **C** and **D**: presence of ductal expression of TGF-ß within invasive ductal carcinoma (C:X200; D:X100). **E** and **F**: presence of stromal expression of TGF-ß R1 within invasive ductal carcinoma (E:X400; F:X100).

Both normal breast ducts (Figure 3A) and tumor epithelial cells expressed (Figures 3C and D) TGF-ß with higher expression in tumor cells compared to normal ducts (p = 0.02). We found no expression of TGF-ß in normal breast tissue and tumor stroma. We found expression of TGF-ßR1 in normal breast ducts and tumor epithelial cells with no statistically significant difference (p = 0.4) (Figure 3B and F). On the other hand, the myofibroblastic stroma of the tumor expressed more TGF- ßR1 than the normal stroma (p = 0.001) (Figures 3E and F). This expression of TGFß-R1 in the tumor stroma was present regardless of the level of expression of TGF-ß by the tumor (Table 4).

**Table 4:** TGF-ß and TGF-ßR1 expression in tumor and normal tissue

	**Tumor tissue**	**Normal tissue**	
Stromal TGF- ß	0	0	p > 0.05
Glandular TGF- ß	185 + − 81	125 + −41	p = 0.02
Stromal TGF- ß R1	197 + −104	80 + −27	p = 0.001
Glandular TGF- ß R1	129 + −102	110 + −22	p = 0.4

Significant p value is <0.05.

### In vitro transformation of fibrocytes into myofibroblasts by TGF-ß1 in mammary cell line fibrocytes

**Figure 4:**
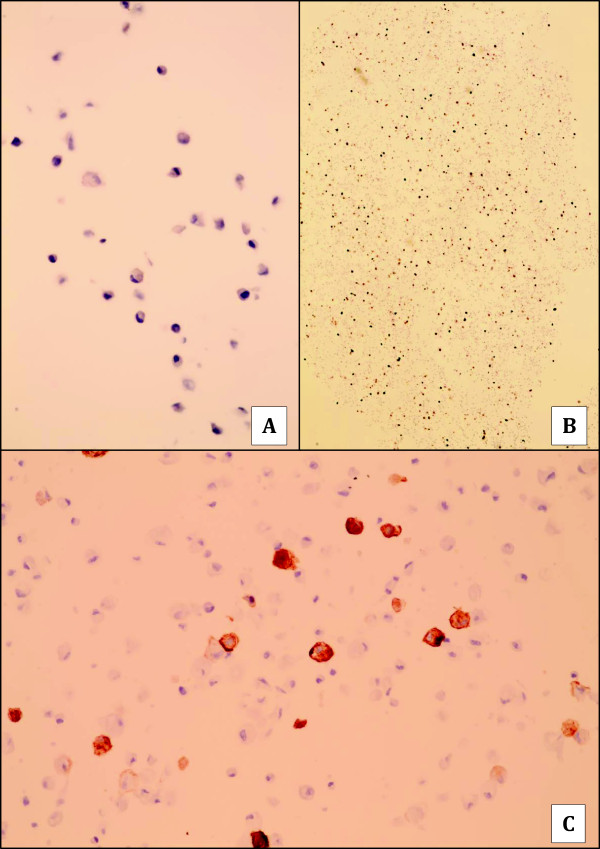
**CD34 and SMA expression in breast fibrocytes cell line before and after treatment by TGF-ß1. A**: absence of an expression of CD34 within untreated breast fibrocytes (X200). **B** and **C**: presence of a significant proportion of SMA-positive myofibroblasts after treatment of breast fibrocytes by TGF-ß1 (B:X40; C:X400).

The proportion of myofibroblasts in each culture treated with 0 or 2.5 ng/ml TGF-ß1 was determined by counting the number of cells immunostained for SMA expression. The percentage of myofibroblast varied from 0 to 70% among the normal cultures and cultures with TGF-ß1 treatment causing a noticeable shift in the percentage of activated myofibroblasts in many normal and treated cultures (Figure 4). TGF-ß1 treatment significantly increased the mean percentage of myofibroblasts in cultures (p < 0.05). It should be noted that the fibrocytes of this cell line do not express CD34.

## Discussion

The importance of changes in the microenvironment during tumor progression has been increasingly recognized [3,15,16]. We have just demonstrated in this work the appearance of a myofibroblastic reaction accompanied by a loss of fibrocytes in IDC. This reaction is present in almost 100% of cases, irrespective of clinical and histological parameters (age, tumor size, tumor shape, grade of invasive carcinoma, luminal classification and KI-67 index). This phenomenon is therefore almost constant and more than likely plays an important tumoral role, particularly in the invasion process. Furthermore and importantly, this pro-invasive action seems to be confirmed by the fact that an intense expression of SMA myofibroblasts was correlated with the presence of lymph node metastasis. In cancer, myofibroblasts may induce the production of proinvasive proteinases [17]. We already carried out a study on the stromal expression of CD34 and SMA in ductal carcinoma in situ (DCIS) [18]. Our in vitro experiments showed a transformation of fibrocytes into myofibroblasts by TGF-ß1, which is one of the main agents involved in this fibro-myofibroblastic transformation. Indeed, different studies have shown that TGF-ß upregulates SMA expression in fibrocytes and transdifferentiates them into myofibroblasts [19,20] This in vitro study also showed, for the first time, that fibrocytes not expressing CD34 are also capable of transforming into myofibroblasts under the action of TGF-ß1. Indeed, in a previous study, we thought that only periductal fibrocytes expressing CD34 were able to transform into SMA myofibroblasts [21]. In our opinion, the loss of fibrocytes and myofibroblast activation does not appear to be just a passive reaction. We believe they are an integral part of the process by facilitating tumor progression and tumor invasion. Besides their role in wound healing, myofibroblasts provide pro-invasive signals that in combination affect invasion of the cancer cells [22,23]. The cross-talk between cancer cells and stromal cells may be mediated through direct heterotypic cell-to-cell contact or through secreted molecules, comprising growth factors, cytokines, chemokines, extracellular matrix proteins, proteinases, proteinase inhibitors, and lipid products [24]. The mechanism leading to the loss of fibrocytes and the appearance of SMA myofibroblasts in the stroma of invasive carcinomas is complex and far from being understood. Breast cancer cells have been shown to be capable of factor secretion [25]. Therefore, we speculate that loss of CD34 fibrocytes and gain of SMA myofibroblasts might be initiated by a soluble factor secreted by tumor cells and especially TGF-ß. We have shown that medium conditioned with TGF-ß1 induces SMA expression in mammary fibrocytes stromal cell line. Moreover, some research has already found that fibrocytes acquire SMA expression when exposed to TGF-ß [19,21]. Considering the results of the present study, it appears to be more likely that mammary fibrocytes acquire SMA having been treated by TGF-ß1. Indeed, our in vivo study of the immunohistochemical expression of TGF-ß and TGF-ßR1 allowed us to show that:

1) Tumor cells secrete TGF-ß and normal fibrocytes have TGF-ß receptors. As demonstrated in vitro, tumor cells are therefore able to transform fibrocytes into SMA myofibroblasts.

2) Tumor cells may have an autocrine effect on their growth because they have TGF-ßR1 and express TFG-ß.

3) Since the stroma does not express TGF-ß, it therefore seems unlikely that the stroma may trigger the process of tumorigenesis via the TGF-ß pathway in any case.

We believe that several mechanisms may explain the promotion of tumor invasion in breast tissue induced by the loss of CD34 fibrocytes and the gain of SMA myofibroblasts.

What are the mechanisms involved in the pro-invasive capacity of fibrocytes?

1) CD34 fibrocytes are potent antigen-presenting cells and might be involved in specific immune surveillance [26,27].

2) CD34 fibrocytes are involved in the remodeling of stromal tissue damage not only through tissue contractility via TGF-ß, collagen I and III synthesis and SMA, but also in terms of migration factors within the injured tissue via CCR7, CXCR4, SLC, and CXCL12.

3) CD34 fibrocytes also play a role in angiogenesis via fibroblast growth factor, vascular endothelial growth factor, platelet-derived growth factor, interleukin-8, and matrix metalloproteinase-9.

What are the mechanisms involved in the pro-invasive capacity of myofibroblasts?

1) The increase in myofibroblasts in breast cancer could result from transdifferentiation of resident interstitial cells expressing or not expressing CD34 fibrocytes into myofibroblasts.

2) Orimo et al. [23] demonstrated that carcinoma-associated fibroblasts (CAF), represented to a large degree by myofibroblasts, promote tumor growth and increase tumor angiogenesis by secretion of stromal derived factor (SDF)-1/CXCL12, which acts in a paracrine fashion to increase tumor cell proliferation via CXCR4. Hepatocyte growth factor (HGF) is another CAF-derived factor that has been implicated in promoting tumor progression and metastasis. The paracrine activation of c-Met on tumor cells by HGF increases invasion of experimental DCIS lesions in xenografts, for example [28]. Interestingly, co-culture of normal mammary fibroblasts with breast cancer cells can ‘educate’ the fibroblasts to secrete HGF and increase their tumor-promoting activities [29].

3) The causal role of myofibroblasts in the transition from the non-invasive towards the invasive phenotype is suggested by the finding that the appearance of myofibroblasts precedes the invasive stage of cancer. This hypothesis seems to be confirmed in one of our previous studies in which we demonstrated the appearance of myofibroblasts around the lesions of DCIS. This expression was more intense around the high-grade lesions (pre-invasive lesions) [18].

4) Associated myofibroblasts prevent physical contact between cancer cells and immune cells, an essential phenomenon for cancer cell destruction. Histology of different types of tumors indicates that, in those tumors in which the myofibroblastic network is poorly developed, inflammatory cells infiltrate the tumors and are in close contact with the cancer cells. In contrast, the presence of myofibroblasts around progressive tumors is associated with the absence of immune and inflammatory cells within tumors [30].

5) In contrast to wound healing, myofibroblasts in the tumor microenvironment do not disappear by apoptosis, indicating that cancer is a wound that does not heal [31].

6) The stromal reaction induced by carcinomatous lesions leads to acquisition of SMA expression and in turn to stabilization of the lesion (wound contraction) that helps prevent the spread of tissue damage [32]. This may reflect a defense mechanism against “stromal invasion” that induces a phenomenon of stromal healing and stabilization. However, the phenotypic transformation or suppression of (CD34) fibrocytes into SMA myofibroblasts could also cause the loss of most essential functions (including immunity, cell adhesion, motility, stromal remodeling, and angiogenesis inhibition), and in a paradoxical manner promote tumorigenesis, thus facilitating invasion and metastatic dissemination of tumor cells.

## Conclusions

The present study demonstrated that a significant tumor myofibroblastic reaction is correlated with the presence of lymph node metastases and that this myofibroblastic reaction can be induced by TGF-ß1. Future larger studies on fibrocytes, myofibroblasts, TGF-ß and stromal change mechanisms are needed to confirm these results and may potentially lead to new treatment approaches.

## Abbreviations

SMA: Smooth muscle actin; ECM: Extracellular matrix; DCIS: Ductal carcinoma in situ; IDC: Invasive ductal carcinoma; TGF-ß: Transforming growth-beta; IPG: Pathology and Genetics Institute; ER: Estrogen receptor; PR: Progesterone receptor; TGF-ßR1: Transforming growth-beta receptor-1; CAF: Carcinoma-associated fibroblasts; HGF: Hepatocyte growth factor.

## Competing interests

The authors declare that they have no competing interests.

## Authors’ contributions

XC, PS and JCN conceived the study and participated in its design. XC and JCN provided formalin-fixed, paraffin-embedded archived patient materials for the study. XC and JCN performed immunostaining. XC, PS and JCN conducted pathological reviews and clinical data evaluations. XC, PS and JCN performed statistical analyses. XC cultured cell lines. XC and JCN conducted in vivo experiments. XC conducted in vitro experiments. XC, PS and JCN drafted the manuscript. All authors read, edited and approved the final manuscript.

## Pre-publication history

The pre-publication history for this paper can be accessed here:

http://www.biomedcentral.com/1471-2407/14/499/prepub

## References

[B1] Shibuya K Mathers CD Boschi-Pinto C Lopez AD Murray CJ Global and regional estimates of cancer mortality and incidence by site: II. Results for the global burden of disease 2000 BMC Cancer 2002 2 37 10.1186/1471-2407-2-37 12502432 PMC149364

[B2] Place AE Jin Huh S Polyak K The microenvironment in breast cancer progression: biology and implications for treatment Breast Cancer Res 2011 13 227 10.1186/bcr2912 22078026 PMC3326543

[B3] Weinberg R Mihich E Eighteenth annual pezcoller symposium: tumor microenvironment and heterotypic interactions Cancer Res 2006 66 11550 11553 10.1158/0008-5472.CAN-06-3149 17158190

[B4] Barth PJ Ebrahimsade S Ramaswamy A Moll R CD34+ fibrocytes in invasive ductal carcinoma, ductal carcinoma in situ, and benign breast lesions Virchows Arch 2002 440 298 303 10.1007/s004280100530 11889601

[B5] Barth PJ Moll R Ramaswamy A Stromal remodeling and SPARC (secreted protein acid rich in cysteine) expression in invasive ductal carcinomas of the breast Virchows Arch 2005 446 532 536 10.1007/s00428-005-1256-9 15838642

[B6] Faler BJ Macsata RA Plummer D Mishra L Sidawy AN Transforming growth factor-beta and wound healing Perspect Vasc Surg Endovasc Ther 2006 18 55 62 10.1177/153100350601800123 16628336

[B7] O’Kane S Ferguson MW Transforming growth factor beta s and wound healing Int J Biochem Cell Biol 1997 29 63 78 10.1016/S1357-2725(96)00120-3 9076942

[B8] Wahl SM Transforming growth factor-beta: innately bipolar Curr Opin Immunol 2007 19 55 62 10.1016/j.coi.2006.11.008 17137775

[B9] Fleisch MC Maxwell CA Barcellos-Hoff MH The pleiotropic roles of transforming growth factor beta in homeostasis and carcinogenesis of endocrine organs Endocr Relat Cancer 2006 13 379 400 10.1677/erc.1.01112 16728569

[B10] Ronnov-Jessen L Petersen OW Induction of alpha-smooth muscle actin by transforming growth factor-beta 1 in quiescent human breast gland fibroblasts. Implications for myofibroblast generation in breast neoplasia Lab Invest 1993 68 696 707 8515656

[B11] Sieuwerts AM Klijn JG Henzen-Logmans SC Foekens JA Cytokine-regulated urokinase-type-plasminogen-activator (uPA) production by human breast fibroblasts in vitro Breast Cancer Res Treat 1999 55 9 20 10.1023/A:1006190729866 10472775

[B12] Roberts AB Sporn MB Assoian RK Smith JM Roche NS Wakefield LM Heine UI Liotta LA Falanga V Kehrl JH Transforming growth factor type beta: rapid induction of fibrosis and angiogenesis in vivo and stimulation of collagen formation in vitro Proc Natl Acad Sci U S A 1986 83 4167 4171 10.1073/pnas.83.12.4167 2424019 PMC323692

[B13] Elston CW Ellis IO Pathological prognostic factors in breast cancer. I. The value of histological grade in breast cancer: experience from a large study with long-term follow-up Histopathology 1991 19 403 410 10.1111/j.1365-2559.1991.tb00229.x 1757079

[B14] Goulding H Pinder S Cannon P Pearson D Nicholson R Snead D Bell J Elston CWE Robertson JF Blamey RW Ellis IO A new immunohistochemical antibody for the assessment of estrogen receptor status on routine formalin-fixed tissue samples Hum Pathol 1995 26 291 294 10.1016/0046-8177(95)90060-8 7890280

[B15] Bissell MJ Kenny PA Radisky DC Microenvironmental regulators of tissue structure and function also regulate tumor induction and progression: the role of extracellular matrix and its degrading enzymes Cold Spring Harb Symp Quant Biol 2005 70 343 356 10.1101/sqb.2005.70.013 16869771 PMC3004779

[B16] Tlsty TD Hein PW Know thy neighbor: stromal cells can contribute oncogenic signals Curr Opin Genet Dev 2001 11 54 59 10.1016/S0959-437X(00)00156-8 11163151

[B17] De Wever O Demetter P Mareel M Bracke M Stromal myofibroblasts are drivers of invasive cancer growth Int J Cancer 2008 123 2229 2238 10.1002/ijc.23925 18777559

[B18] Catteau X Simon P Vanhaeverbeek M Noel JC Variable stromal periductular expression of CD34 and smooth muscle actin (SMA) in intraductal carcinoma of the breast PLoS One 2013 8 e57773 10.1371/journal.pone.0057773 23469238 PMC3585862

[B19] Abe R Donnelly SC Peng T Bucala R Metz CN Peripheral blood fibrocytes: differentiation pathway and migration to wound sites J Immunol 2001 166 7556 7562 10.4049/jimmunol.166.12.7556 11390511

[B20] Allinen M Beroukhim R Cai L Brennan C Lahti-Domenici J Huang H Porter D Hu M Chin L Richardson A Schnitt S Sellers WR Polyak K Molecular characterization of the tumor microenvironment in breast cancer Cancer Cell 2004 6 17 32 10.1016/j.ccr.2004.06.010 15261139

[B21] Espana EM Kawakita T Liu CY Tseng SC CD-34 expression by cultured human keratocytes is downregulated during myofibroblast differentiation induced by TGF-beta1 Invest Ophthalmol Vis Sci 2004 45 2985 2991 10.1167/iovs.04-0201 15326111

[B22] Powell DW Mifflin RC Valentich JD Crowe SE Saada JI West AB Myofibroblasts. I. Paracrine cells important in health and disease Am J Physiol 1999 277 C1 C9 10409103 10.1152/ajpcell.1999.277.1.C1

[B23] Orimo A Tomioka Y Shimizu Y Sato M Oigawa S Kamata K Nogi Y Inoue S Takahashi M Hata T Muramatsu M Cancer-associated myofibroblasts possess various factors to promote endometrial tumor progression Clin Cancer Res 2001 7 3097 3105 11595701

[B24] De Wever O Mareel M Role of tissue stroma in cancer cell invasion J Pathol 2003 200 429 447 10.1002/path.1398 12845611

[B25] Liang X Huuskonen J Hajivandi M Manzanedo R Predki P Amshey JR Pope RM Identification and quantification of proteins differentially secreted by a pair of normal and malignant breast-cancer cell lines Proteomics 2009 9 182 193 10.1002/pmic.200700957 19053080

[B26] Sircar K Hewlett BR Huizinga JD Chorneyko K Berezin I Riddell RH Interstitial cells of Cajal as precursors of gastrointestinal stromal tumors Am J Surg Pathol 1999 23 377 389 10.1097/00000478-199904000-00002 10199467

[B27] Suster S Fisher C Immunoreactivity for the human hematopoietic progenitor cell antigen (CD34) in lipomatous tumors Am J Surg Pathol 1997 21 195 200 10.1097/00000478-199702000-00009 9042286

[B28] Jedeszko C Victor BC Podgorski I Sloane BF Fibroblast hepatocyte growth factor promotes invasion of human mammary ductal carcinoma in situ Cancer Res 2009 69 9148 9155 10.1158/0008-5472.CAN-09-1043 19920187 PMC2789178

[B29] Tyan SW Kuo WH Huang CK Pan CC Shew JY Chang KJ Lee EY Lee WH Breast cancer cells induce cancer-associated fibroblasts to secrete hepatocyte growth factor to enhance breast tumorigenesis PLoS One 2011 6 e15313 10.1371/journal.pone.0015313 21249190 PMC3020942

[B30] Opdenakker G Van Damme J Chemotactic factors, passive invasion and metastasis of cancer cells Immunol Today 1992 13 463 464 10.1016/0167-5699(92)90079-M 1298279

[B31] Dvorak HF Tumors: wounds that do not heal. Similarities between tumor stroma generation and wound healing N Engl J Med 1986 315 1650 1659 10.1056/NEJM198612253152606 3537791

[B32] Satish L Gallo PH Baratz ME Johnson S Kathju S Reversal of TGF-beta1 stimulation of alpha-smooth muscle actin and extracellular matrix components by cyclic AMP in Dupuytren’s-derived fibroblasts BMC Musculoskelet Disord 2011 12 113 10.1186/1471-2474-12-113 21612641 PMC3125251

